# Elements of patient satisfaction: An integrative review

**DOI:** 10.1002/nop2.1437

**Published:** 2022-10-28

**Authors:** George W. Goodrich, James Mark Lazenby

**Affiliations:** ^1^ University of Connecticut School of Nursing Storrs Connecticut USA; ^2^ University of California Irvine Sue & Bill Gross School of Nursing Irvine California USA

**Keywords:** integrative review, measure development, nursing, patient satisfaction, quality of nursing care

## Abstract

**Aim:**

To summarize the scientific literature on the elements essential to understanding a nursing definition of patient satisfaction.

**Design:**

Whittemore and Knafl's methodology was used for this integrative review.

**Methods:**

Articles were included if the studies they explored patient satisfaction in patient populations and measured patient satisfaction using standardized, validated instruments. Elements in this review were defined as the essential components that create the complex concept of patient satisfaction.

**Results:**

Thirty articles were found and analysed in full. Five definitions of patient satisfaction were used, all of which were at least 20 years old. Twenty‐two different measures of patient satisfaction were used, six of which were nursing‐specific. Sixty‐eight elements of patient satisfaction were studied in the included articles. Forty‐three elements were reported as having a significant relationship with patient satisfaction, 25 were reported as having no significant relationship. Eight elements had both significant and non‐significant relationships.

## 1 INTRODUCTION

Nurses have a major role in creating patient satisfaction. Butler et al. (2018) found critical care nurses spent 44.3% of their daytime working hours in or immediately outside a patient's room. This is more than double the time of physicians (14.7%), and other critical staff, including respiratory and physical therapists (20.5%). When patients were receiving direct care, 86.1% of the time it was with a nurse (Butler et al., 2018). A large portion of patient satisfaction may well be formed while under a nurse's direct care. The standardized evaluation of patient satisfaction in the United States, through Press Ganey and the introduction of the Hospital Consumer Assessment of Healthcare Providers and Systems (HCAHPS) in 2006, has changed how nursing performance is measured. The problem of standardization is not just within the United States, however; there is no single globally agreed‐upon definition or measure of patient satisfaction. Understanding the elements of patient satisfaction can help researchers, practitioners and administrators focus their efforts on where nurses can actually make a difference in patient satisfaction.

## 2 BACKGROUND

To improve nursing care and patient outcomes, researchers have been working to define patient satisfaction and create measures to quantify it for more than five decades (Abdellah & Levine, 1957; Bernays, 1947; Copp, 1971; McGhee, 1961: Ozturk et al., 2020). In the United States (U.S.), patient satisfaction surveys are currently used to evaluate the quality of healthcare received by the patient and nursing job performance and as part of the Medicare formula for hospital reimbursement (Petrullo et al., 2012). Hospitals that receive Medicare funding are required to participate in HCAHPS. More than 4,000 community hospitals in the United States participated in the HCAHPS survey programme in 2020 (Centers for Medicare and Medicaid Services, 2020a). The results of HCAHPS are shared publicly to aid consumers in healthcare decisions. In addition, Press Ganey surveys 40 million patients annually about their experience with different types of healthcare providers (Press Ganey, 2021). Press Ganey satisfaction surveys, while not shared publicly, are frequently used to evaluate nursing care on a unit level and can be part of nursing manager performance reviews. Hospital administrators often allocate resources and set nursing policy based on the findings from these patient satisfaction surveys (Gray et al., 2016).

Hospital Consumer Assessment of Healthcare Providers and Systems uses a 27‐question self‐reported feedback survey to gather data from patients who have been discharged. There is particular emphasis placed on the question of whether the patient would “recommend this hospital to your friends and family” (Centers for Medicare and Medicaid Services, 2020b, p. 323). Press Ganey uses similar questions and emphasis. The “would you recommend” question is used more broadly in marketing to measure brand loyalty and predict company growth (Reichheld, 2003). Using it in the healthcare setting, however, assumes that customer loyalty is equivalent to patient satisfaction. This points to a gap. A nursing‐specific measure of patient satisfaction would measure the patient's perspective of nursing care, as opposed to their perspectives on marketing or the hospital environment, over which nurses have little or no influence.

The concept of patient satisfaction, despite the five decades of work, lacks an agreed‐upon definition (Turris, 2005; Wolf et al., 2014). Without an explicit definition, it would be difficult to determine elements, that is, the conceptual content (Audi, 2015), of patient satisfaction. It would likewise be difficult (if not impossible) to measure patient satisfaction without conceptual content. Elements are the building blocks of a concept, without which, the concept would not be complete. A myriad of elements of care could influence a patient's perception of satisfaction, from food choices to waiting times to the personalities of caregivers and patients themselves. However, given the weight placed upon patient satisfaction, it is important to summarize the scientific literature vis‐à‐vis the elements of the concept of patient satisfaction.

The purpose of this integrative review was to summarize the scientific literature on the elements essential to understanding a nursing definition of patient satisfaction.

## 3 STUDY

### 3.1 Design

Whittemore and Knafl's (2005) methodology was used for this integrative review. The criteria for reporting this review followed the guidelines of the Preferred Reporting for Systemic Reviews and Meta‐Analysis (PRISMA).

### 3.2 Methods

#### 3.2.1 Literature search

The literature search was conducted from November–December of 2020 using Cumulative Index to Nursing and Allied Health Literature (CINAHL) and PubMed databases. The search was updated on June 26, 2021, where no additional articles were found that fit the inclusion criteria. Table 1 presents the search terms and Boolean operators used in the search strategy, which were guided by a nursing librarian.

**TABLE 1:** Boolean operator terms

Search #	Terms
1	patient satisfaction AND mapping analysis
2	patient satisfaction AND (definition or define or meaning or description)
3	patient satisfaction AND (definition or define or meaning or description) AND (inpatients or hospitalized patients or hospitalized patients)
4	TI define patient satisfaction OR AB define patient satisfaction
5	(MH “Patient Satisfaction+”) AND (MH “Nursing Care+”)
6	(MH “Patient Satisfaction+”) AND (MH “Nursing Care+”)
7	((MH “Patient Satisfaction+”) AND (MH “Nursing Care+”)) AND nursing patient relationship
8	TI Measuring Satisfaction
9	TI Improving Patient Satisfaction
10	Patient Satisfaction AND Nursing Role
11	Patient Satisfaction AND Nursing
12	Patient Satisfaction AND Nursing Satisfaction
13	Uncaring AND Nursing
14	Effects of nursing care on patient satisfaction

#### 3.2.2 Inclusion and exclusion criteria

Articles were included if the studies explored patient satisfaction in patient populations and measured patient satisfaction using standardized, validated instruments and were published in peer‐reviewed journals between 2000–2021 in English. The dates for inclusion were chosen to capture a significant change in how patient satisfaction was measured in the United States with the introduction of HCAHPS in 2006.

Studies were excluded if they were case reports, literature and systemic reviews, opinion/editorial articles or observational studies.

#### 3.2.3 Procedures

Each article was screened by title and abstract for the inclusion and exclusion criteria by the first author. Both authors screened articles that fit the criteria by full text. Disagreements whether to include articles were resolved by discussion and consensus between the authors. During this process the first author reviewed references of the considered articles for additional studies that fit the criteria. These articles were then subjected to review by both authors as above.

#### 3.2.4 Quality appraisal

The quality assessment of the included studies followed two criteria, methodological or theoretical rigour and data relevance, on a 2‐point scale (high = 2, low = 1) (Whittemore & Knafl, 2005). No studies were excluded on the basis of quality; however, during the analysis, more weight was given to studies with rigour and data relevance rates of 2.

### 3.3 Data extraction, analysis and synthesis

The purpose of this integrative review was to summarize the scientific literature on the elements essential to a nursing definition of patient satisfaction.

In this review, data were defined as results of studies that measured patient satisfaction; identified elements or factors that are not elements, of patient satisfaction; and written experiences of patient satisfaction. All studied elements were included if they had a significant relationship with patient satisfaction (*p* < .05) or if they were rejected. The exception to this was the one qualitative article included, where the authors' interpretations were reported.

**FIGURE 1:**
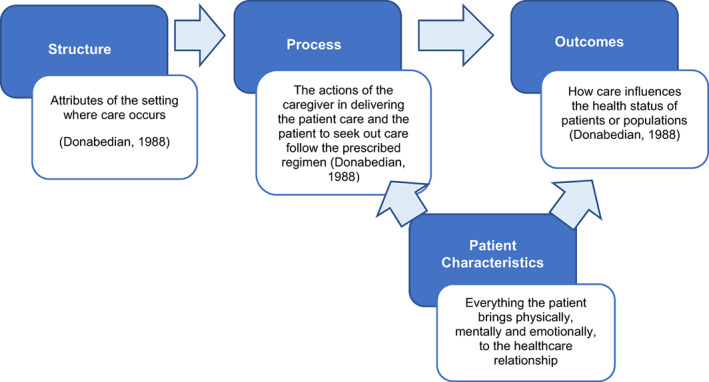
Donabedian's model modified to include patient characteristics

Donabedian's conceptual model of assessing healthcare quality was used to organize the elements into the subcategories of structure, process and outcome (Donabedian, 1966). Kurowski and Shaughnessy (1982) posit patient characteristics can influence both process and outcomes for individuals. We modified Donabedian's model to include patient characteristics as a subcategory of patient satisfaction elements (Figure 1).

Elements in this review were defined as the essential components that create the complex concept of patient satisfaction. Each identified element was compared, and a determination was made if the elements were essentially the same or if there was enough uniqueness to categorized them separately.

The results of each study were entered into a data extraction tool and a coding index within Microsoft Word documents. The data extraction tool identified each article's research question, study design, setting, population, definition and components, measures used, intervention, results and the study's strengths and limitations. The first author organized the data into categories based on an extended Donabedian model. Both authors examined the data for patterns and relationships that might give a better understanding of the elements that make up patient satisfaction. Findings are the authors' interpretation of the data.

### 3.4 Ethical considerations

Because this review did not include human participants and only previously published research, no patient consent or ethical approval was sought.

## 4 RESULTS

**FIGURE 2:**
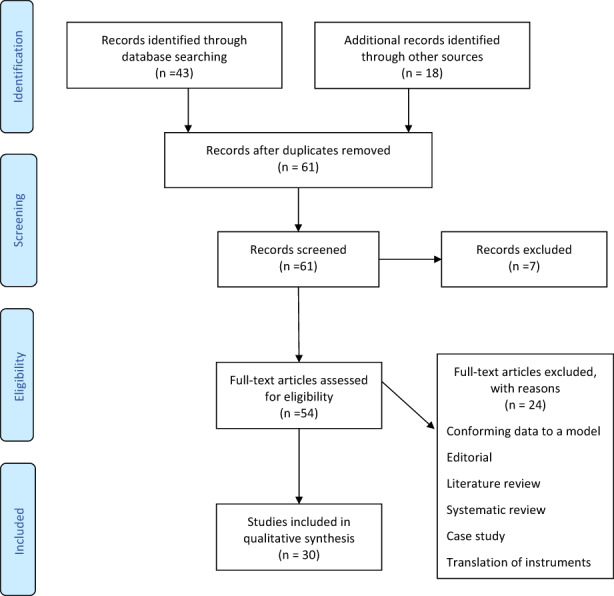
PRISMA flowsheet

Thirty articles were included in the analysis. Figure 2 presents the PRISMA flow sheet.

### 4.1 Study characteristics

Of the 30 articles included for review, 13 reported on studies conducted exclusively in the United States. Two articles reported on studies that were conducted in both Norway and Turkey. France, Iran, Jordan, Malaysia, Netherlands, Poland, Slovenia, South Africa, Spain, Sweden and Taiwan were represented by one article each. One article reported on a study that included the United States and 12 European countries. Another article included five European countries.

Study designs varied in the selected articles, but most were cross‐sectional (*n* = 17). Other designs included prospective cohort studies (*n* = 4) and retrospective cohort studies (*n* = 3). Instrument development, randomized mode experiment, theoretical model testing, quasi‐experimental, descriptive comparative and qualitative study designs were all represented by one article. The reviewed articles overwhelmingly studied inpatient populations (*n* = 23), but outpatient settings were represented (*n* = 5), and two studies did not differentiate (Flood et al., 2016; Polak et al., 2019).

### 4.2 Definition of patient satisfaction

Five of the articles presented one or more definitions of patient satisfaction, yet none was original to the author (Table S1). The oldest definition found in the included literature was from 1975, and the most current definition from 2001.

Four of the five definitions describe patient satisfaction as a reaction to, or outcome of, an interaction between patients and healthcare providers (Findik et al., 2010; Larrabee, 2003; Mrayyan, 2006; Suhonen et al., 2012). Patient satisfaction, in these definitions, is a post‐care assessment. The fifth defines patient satisfaction as a measure of the current state of the relationship between healthcare providers and healthcare consumers (Ríos‐Risquez & García‐Izquierdo, 2016). That is, rather than a post‐care assessment of satisfaction, or an outcome, it is an in‐the‐middle‐of‐care assessment, identifying it as a process.

### 4.3 Measuring patient satisfaction

Twenty‐two instruments were used to measure patient satisfaction in the included articles. HCAHPS was used by six articles, Press Ganey satisfaction scores were used in three, and the Patient Perception of Hospital Experience with Nursing (PPHEN) was also used in three articles. No other instruments were used more than once (Table S2).

Nine of the 14 articles (64%) written in the United States used either HCAHPS or Press Ganey to measure patient satisfaction. Three articles written in the United States before HCAHPS was implemented (2006) used PPHEN (Dozier et al., 2001), Patient's Evaluation of Performance in California (PEP‐C) (Burns‐Bolton et al., 2003) or the Patients' Judgements of Nursing Care (Larrabee et al., 2004). The two articles written in the United States after 2006 that did not use HCAHPS or Press Ganey used an adaptation of the Army Provider Level Satisfaction Scale (APPLS) survey (Dragovich et al., 2017) and the Medical Expenditure Panel Survey Household Component (MEPS‐HC) (Chen et al., 2018). The PPHEN survey was used twice in articles from beyond the United States. Every other article used a different instrument to measure patient satisfaction.

### 4.4 Elements of care related to patient satisfaction

Collectively, articles reported on 43 elements with a related to patient satisfaction and 25 element that were not related with patient satisfaction. Reported elements with significant relationships with patient satisfaction can be found in Table 2. Elements without significant relationships with patient satisfaction are found in Table 3.

**TABLE 2:** Elements found to be related to patient satisfaction

#	Element	Article
*Structure elements of patient satisfaction*		
1	Total nursing hours per patient day	Burns‐Bolton et al. (2003)
2	Coordination of care	Yen and Lo (2004)
3	perceived quality of nursing care	Yen and Lo (2004)
4	Hospital Size^a^ (Large and Teaching Hospitals had negative relationship)	Sjetne et al. (2007)
	Larger hospitals had positive relationship	Kennedy et al. (2014)
5	Length of hospital stay^a^ (longer being more satisfied)	Findik et al. (2010)
6	Hospitals with good work environments (for nurses)	Aiken et al. (2012)
7	Nurse Staffing	Aiken et al. (2012), Peršolja (2018)
8	Individualized Care (moderate effect)	Suhonen et al. (2012)
9	Low Hospital Mortality Index	Kennedy et al. (2014)
10	Higher Surgical Volume (Positive relationship)	Kennedy et al. (2014)
11	Cleanliness of Hospital environment	Westbrook et al. (2014)
12	Door‐to‐Provider times (week inverse relationship)	Flood et al. (2016)
13	Wait times in registration	Godley and Jenkins (2019)
14	Wait times in test/treatment	Godley and Jenkins (2019)
*Process elements of patient satisfaction*		
15	Patient Perceived Nurse Care	Larrabee et al. (2004)
16	Nurses' job satisfaction	Mrayyan (2006)
17	Quality of nursing care	Mrayyan (2006)
18	Communication with nurses	Westbrook et al. (2014)
19	Communication with Doctors	Westbrook et al. (2014)
20	Communication about medications	Westbrook et al. (2014)
21	Responsiveness of hospital staff	Westbrook et al. (2014)
22	Being carefully listened to by the provider	Dragovich et al. (2017)
23	Being treated with courtesy and respect	Dragovich et al. (2017)
24	Perception of being helped	Dragovich et al. (2017)
25	Co‐constructed interaction	Mohammadipour et al. (2017)
*Patient characteristics of patient satisfaction*		
26	Age^a^ (Positive relationship)	Westaway et al. (2003) Larrabee et al. (2004) Yen and Lo (2004) Hekkert et al. (2009) Larsson and Wilde‐Larsson (2010) Findik et al. (2010) Nguyen et al. (2011)
	Age (Inverse relationship)	Chen et al. (2018) Ozturk et al. (2020)
27	Mental Health Status^a^	Westaway et al. (2003) Larsson and Wilde‐Larsson (2010) Chen et al. (2018)
28	Interpersonal Subscale	Westaway et al. (2003)
29	Sex^a^ (there is a difference)	Foss and Hofoss (2004)
	(Male higher satisfaction)	Findik et al. (2010)
	(Female higher satisfaction)	Chen et al. (2018) Hekkert et al. (2009)
30	Quality of Life	Larrabee et al. (2004)
31	Education (positive relationship)	Hekkert et al. (2009) Ozturk et al. (2020)
32	Self‐Reported Health Status^a^	Hekkert et al. (2009), Larsson and Wilde‐Larsson (2010), Nguyen et al. (2011)
33	Native Born	Larsson and Wilde‐Larsson (2010), Tang et al. (2013)
34	Viewed doctors favourably	Larsson and Wilde‐Larsson (2010)
35	Income (negative relationship)	Findik et al. (2010)
	Income (positive relationship)	Ozturk et al. (2020)
36	Radiotherapy (more negative) vs Chemotherapy	Nguyen et al. (2011)
37	Head and Neck Cancer (more negative) vs other cancer sites	Nguyen et al. (2011)
38	Patient level of stress	Dragovich et al. (2017)
39	Race (Black/African American inverse relationship)	Chen et al. (2018)
40	Insurance (Medicaid inverse relationship)	Chen et al. (2018)
41	Socioeconomic status (inverse relationship)	Chen et al. (2018)
42	More than 2 ED visits	Chen et al. (2018)
43	Employment	Ozturk et al. (2020)

^a^ Elements found to have both a significant and non‐significant relationship with patient satisfaction.

**TABLE 3:** Elements found not to be related to patient satisfaction

#	Element	Article
*Process elements not related to patient satisfaction*		
1	Hospital size^a^	Burns‐Bolton et al. (2003)
2	Total caregiver hours per patient	Burns‐Bolton et al. (2003)
3	Percent RN Hours	Burns‐Bolton et al. (2003)
4	Percent contracted hours	Burns‐Bolton et al. (2003)
5	Safety Indicators	Kennedy et al. (2014)
6	Length of stay^a^	Kennedy et al. (2014)
7	Complications	Kennedy et al. (2014)
8	Readmission	Kennedy et al. (2014)
9	Type of treatment (medical/surgical)	Ozturk et al. (2020)
10	Number of days hospitalized	Larrabee et al. (2004)
11	Unit turbulence	Larrabee et al. (2004)
12	Percent of budgeted RN FTEs filled	Larrabee et al. (2004)
13	Ratio of patients to RN matched to patient days of hospitalization	Larrabee et al. (2004)
14	Ratio of RNs to all nursing staff	Larrabee et al. (2004)
15	Aggregated RN job satisfaction	Larrabee et al. (2004)
16	Nurse manager leadership style	Larrabee et al. (2004)
17	Quietness Hospital environment	Westbrook et al. (2014)
*Process elements not related to patient satisfaction*		
18	Discharge information	Westbrook et al. (2014)
19	Nurse Stress Perceptions	Ríos‐Risquez and García‐Izquierdo (2016)
20	Nurse Burnout dimensions (emotional exhaustion, cynicism, personal effectiveness)	Ríos‐Risquez and García‐Izquierdo (2016)
*Patient characteristics elements not related to patient satisfaction*		
21	Self‐Reported Physical Health^a^	Westaway et al. (2003), Chen et al. (2018)
22	Age^a^	Tang et al. (2013)
23	Sex^a^	Tang et al. (2013), Ozturk et al. (2020)
24	Marital status	Tang et al. (2013), Ozturk et al. (2020)
25	Mental Health Status^a^	Larrabee et al. (2004)

^a^ Elements found to have both a significant and non‐significant relationship with patient satisfaction.

#### 4.4.1 Structure

Within the subcategory of structure, 14 elements were found to have a relationship with patient satisfaction, whereas 17 studied elements were found not to be related.

##### Waiting times

Waiting times are an often‐studied element of patient satisfaction. Decreases in waiting times for registration and tests/treatments in an outpatient vascular procedure unit were related to an increase in patient satisfaction (Godley & Jenkins, 2019). Yet, Flood et al. (2016) found that a decrease in door‐to‐provider time in a paediatric emergency room had a weak, inverse relationship with patient satisfaction.

##### Hospital size

Hospital size was found to be an element of patient satisfaction in two articles. Kennedy et al. (2014) found that larger hospitals in the United States were positively related to patient satisfaction. However, Sjetne et al. (2007) found that large and teaching hospitals in Norway were negatively related to patient satisfaction. In 2001, Burns‐Bolton found no relationship between hospital size and patient satisfaction in the California hospitals she studied (Burns‐Bolton et al., 2003).

##### Nurse staffing

A relationship between nurse staffing and patient satisfaction was reported by Aiken et al. in 2012 and again by Peršolja in 2018. Aiken also found hospitals with a good nursing work environment (better nurse to patient ratios, nurses involved in decision making, positive nurse/doctor relations) also had higher patient satisfaction in both the United States and the studied European countries (Aiken et al., 2012). Burns‐Bolton et al. (2003) determined there was a relationship between total nursing hours per patient day and patient satisfaction. The same study also found total nursing hours per patient, percent RN hours (vs nurse assistant hours) and the percent of contracted nurse hours had no relationship with patient satisfaction (Burns‐Bolton et al., 2003). Larrabee et al. (2004) also found no relationship between the percent of RN hours and the number of nursing FTEs budgeted, unit turbulence and the nurse/patient ratio.

##### Patient safety

In studying how patient safety measures effected patient satisfaction, Kennedy et al. (2014) found a lower hospital mortality index, and higher surgical volume related to patient satisfaction. They also found no relationship between safety indicators, complications, length of stay or readmissions and patient satisfaction.

##### Other process elements

Findik et al. (2010) found that a longer length of stay for the patient was related to higher patient satisfaction scores, whereas Larrabee et al. (2004) found no relationship with length of stay. Taiwanese patients gave higher patient satisfaction scores when care was coordinated, and they perceived a high quality of nursing care (Yen & Lo, 2004). Suhonen et al. (2012) found when patients perceived they were receiving individualized care, there was a moderate relationship to higher patient satisfaction. Finally, Westbrook et al. (2014) found a relationship between the cleanliness of the hospital environment and higher patient satisfaction, but found no relationship with the quietness of the hospital environment.

#### 4.4.2 Process

Eleven process elements were identified in the studied literature as related to patient satisfaction, and three studied elements were not related.

##### Communication

Communication between caregivers and patients was a prevalent element of patient satisfaction. Communication with nurses, communication with doctors and communication about medications (Westbrook et al., 2014) were found to correlate with higher patient satisfaction. Similarly, being carefully listened to by caregivers, being treated with courtesy and respect (Dragovich et al., 2017) and the responsiveness of hospital staff (Westbrook et al., 2014) also related to patient satisfaction.

In a qualitative study, Mohammadipour et al. (2017) found five elements of patient satisfaction based on nursing interactions: informed concentration, task‐centred vs patient‐centred relationship, comprehensive participation, accountable encounter and clarification of meanings. The authors combine these elements under the category of co‐constructed interaction, where the patient and nurse interact to create a caring relationship.

##### Patient perceptions

Patient satisfaction was also related to the patients' perceptions of nursing care quality (Larrabee et al., 2004; Mrayyan, 2006) and the patients' perception of being helped by caregivers (Dragovich et al., 2017).

Discharge information, as measured by HCAHPS, was not related to patient satisfaction (Westbrook et al., 2014), and Ríos‐Risquez and García‐Izquierdo (2016) found that a nurse's perception of stress and burnout was also not related to patient satisfaction.

#### 4.4.3 Patient characteristics

Eighteen patient characteristics identified in the literature as elements of patient satisfaction, and five were found to have no relationship.

##### Age

Older patients were found to be more satisfied with their care in eight studies (Chen et al., 2018; Findik et al., 2010; Hekkert et al., 2009; Larrabee et al., 2004; Larsson & Wilde‐Larsson, 2010; Nguyen et al., 2011; Westaway et al., 2003; Yen & Lo, 2004), while Ozturk et al. (2020) found an inverse relationship between age and patient satisfaction, and Tang et al. (2013) found no relationship.

##### Sex

A number of articles found sex to be an element of patient satisfaction. Women had higher reported satisfaction than men in articles by Chen et al. (2018) and Hekkert et al. (2009), while men had higher reported satisfaction than women in an article by Findik et al. (2010). Foss and Hofoss (2004) found women were more likely to fill in free text comments within a multiple‐choice survey than men, and those comments were generally more negative than the multiple‐choice answers given by the same person. Articles by Tang et al. (2013) and Ozturk et al. (2020) found no relationship between sex and patient satisfaction.

##### Health status

A patient's self‐reported physical health status was found to be related with patient satisfaction (Hekkert et al., 2009; Larsson & Wilde‐Larsson, 2010; Nguyen et al., 2011) as was a patient's self‐reported mental health status (Chen et al., 2018; Larsson & Wilde‐Larsson, 2010; Westaway et al., 2003). Westaway et al. (2003) and Chen et al. (2018), on the other hand, found no relationship with self‐reported physical health status, and Larrabee et al. (2004) found no relationship with self‐reported mental health status but did find a relationship with self‐reported quality of life.

##### Other patient feature elements

Education had a positive relationship with patient satisfaction in articles by Hekkert et al. (2009) and Ozturk et al. (2020). Ozturk et al. (2020) also found positive relationships between patient satisfaction and a patient's employment status and income in Turkey. Findik et al. (2010) found an inverse relationship between income and patient satisfaction, also in Turkey.

In an article from the United States, being African‐American, having Medicaid (a government insurance for people with low income) and socioeconomic status were all found to have relationships with lower patient satisfaction (Chen et al., 2018). Patients who were native born to Sweden and Malaysia had higher patient satisfaction than patients who were immigrants or visiting (Larsson & Wilde‐Larsson, 2010; Tang et al., 2013).

In France, patients receiving radiotherapy treatments for cancer and patients with head and neck cancers had lower patient satisfaction scores (Nguyen et al., 2011). Patients who viewed doctors more favourably or were extraverted and emotionally stable had higher patient satisfaction (Larsson & Wilde‐Larsson, 2010). Patients with higher levels of stress (Dragovich et al., 2017) and those who had more than two emergency room visits in the last year (Chen et al., 2018) had lower patient satisfaction scores.

#### 4.4.4 Outcomes

Eight outcomes were identified in the literature where patient satisfaction related to the health status of patients (Table S3). Patient satisfaction with inpatient care and discharge planning were related to a decreased risk of readmission within 30 days (Boulding et al., 2011). Fenton et al. (2012) found increased patient satisfaction related to lower odds of an emergency department visit over 1 year. Yet the same study found that the highest levels of patient satisfaction were related to higher odds of inpatient admission, greater total healthcare expenditures, greater prescription expenditures and higher mortality over 1 year (Fenton et al., 2012). Higher patient satisfaction, as measured by Press Ganey, related to patient loyalty (Kessler & Mylod, 2011) while overall hospital patient satisfaction as measured by HCAPS was related to the willingness of the patient to recommend the hospital to family or friends (Westbrook et al., 2014).

## 5 DISCUSSION

### 5.1 Major findings

The purpose of this integrative review was to summarize the scientific literature on the elements essential to a nursing definition of patient satisfaction.

Only five of the 30 included articles offered a definition of patient satisfaction, and each was at least two decades old. Sixty‐eight elements were identified in the articles reviewed; 43 were found to have a relationship with patient satisfaction; and 25 were found not to have a relationship with patient satisfaction. Eight of the identified elements (12%) were found both to have a relationship and not have a relationship with patient satisfaction. These results suggest that, after 50 years of studying patient satisfaction, there is not an agreed‐upon definition of patient satisfaction nor of conceptual elements. These findings correspond with those of Turris (2005) and Wolf et al. (2014).

#### 5.1.1 Definitions

Donabedian (1992) posits that consumers, or patients, should make a decisive contribution to define what patient satisfaction is. They alone can give certain information such as pain evaluation, expectations, anxieties and physical function to assist care. Even though patients may not have express knowledge of what technical care involves, technical care, according to Donabedian, is delivered via interpersonal exchange. While the five definitions of patient satisfaction take into account the patient's expectations and perceptions, only the definition by Hendriks et al. (2001), as cited by Ríos‐Risquez and García‐Izquierdo (2016), speaks to the exchange between professional caregiver and patient. A nursing definition of patient satisfaction needs to include this interpersonal exchange.

#### 5.1.2 Elements of patient satisfaction

That 68 elements of patient satisfaction were identified indicates the complexity of patient satisfaction as a concept. Increasing the complexity, eight of the identified elements (11.7%) had a positive, negative or no relationship with patient satisfaction, depending on the study. Patient sex, age, length of hospital stays, hospital size, the number of hours spent with an RN, physical health and mental health status all had contradictory results within the articles reviewed.

There are certainly elements of patient satisfaction that have not yet been identified or fully refined. An example of this comes from Kennedy et al. (2014), who found that lower hospital mortality and higher surgical volume in hospitals were related to higher patient satisfaction in U.S. hospitals. Lower hospital morality and higher surgical volume could be complex constructs of their own with nursing staff experience being an element of them. If so, this would cohere with the intersecting definitional elements of patient satisfaction with the structure and process of care.

##### Structure elements

This review found 14 structure elements that have relationships with patient satisfaction (32.5%). Nurses are not often included in developing their workplace. Yet a work environment that is supportive of nursing may allow them the time to communicate with patients and perform technical care that is individualized and not rushed (Aiken et al., 2012; Burns‐Bolton, 2003; Larrabee et al., 2004). Nurses must have more input in the decisions that shape the structure elements of patient satisfaction if they are to be evaluated by patient satisfaction.

##### Process elements

Where nurses do have some control over elements of patient satisfaction is found in process. Of the 43 elements in this review found to have a relationship with patient satisfaction, 11 (25.5%) were process elements. Seven of these 11 elements were based on communication between the caregiver and patient (Dragovich et al., 2017; Mohammadipour et al., 2017; Westbrook et al., 2014). These elements speak to the interpersonal exchange between nurse and patient during the course of treatment.

Structure, according to Donabedian, “increases the likelihood of good process” (Donabedian, 1988, p. 1745). If, as Butler et al. (2018) suggests, nurses give the vast majority of in‐hospital treatments, the workplace needs to offer nurses the chance for their process, the interpersonal exchange involved in treatments, to occur on a routine basis. This will help turn the focus from task‐centred care to patient‐centred care and increase the chance for better patient outcomes and patient satisfaction.

##### Patient characteristic elements

Eighteen of the 43 elements (41.8%) identified as related to patient satisfaction were patient characteristics. A bedside nurse is unable to change most patient characteristics such as age, income, insurance coverage, race or disease process.

Culture and socioeconomic status as elements of patient satisfaction were not directly studied in any of the articles, though they were addressed obliquely. Being native born in Sweden or Malaysia was related to higher patient satisfaction (Larsson & Wilde‐Larsson, 2010; Tang et al., 2013). In the United States, being African‐American, on Medicaid or having a lower socioeconomic status was related to lower patient satisfaction (Chen et al., 2018). These results hint at even more complex and individualized concept of patient satisfaction. If patient characteristics influence care process and outcomes, culture and socioeconomic status need to be included among those characteristics.

#### 5.1.3 Outcomes

Eight patient outcomes related to patient satisfaction were identified in the included articles. All of the articles looking at patient outcomes were written in the United States, used HCAHPS or Press Ganey as a measure and were based on economic or marketing goals. If research began with a nursing‐specific definition of patient satisfaction, and outcomes were also nursing‐specific, researchers would have an easier time addressing the elements of patient satisfaction that are within the nurse's ability to change.

Fenton et al. (2012) found that higher patient satisfaction is related to greater risk of mortality, inpatient admission and increased healthcare and medication expenditures. The authors suggest that an overemphasis on patient satisfaction may take away from efforts to use evidence‐based and patient‐centred care and lead to unintended consequences in patient expenditures and healthcare usage (Fenton et al., 2012). Donabedian also warned against substituting more immediate, visible aspects of care over long‐term, less noticeable care to create more “pleasant circumstances” (Donabedian, 1992, p. 248).

#### 5.1.4 Instruments used to measure patient satisfaction

Most researchers in the United States seem to have defaulted to using the marketing‐based questionnaires of Press Ganey or HCAHPS, even though at least six valid nursing‐specific patient satisfaction measures exist. This could be, in part, due to ease of use. HCAHPS is already required for most hospitals and the results easily accessed, so no new survey is required.

Yet, there are limits to HCAHPS and Press Ganey. They focus on the question of whether the patient “would … recommend” the hospital, provider or service. This type of question was created as a marketing metric of customer loyalty. While HCAHPS and Press Ganey both ask about interactions with nurses, they are not a nursing‐specific measure looking for nursing‐specific outcomes.

Six nursing‐specific instruments were used to measure patient satisfaction (Table S2). Five of these instruments, PPHEN, NSNCS, PSNCS, The Satisfaction with Nursing Care and La Monica‐Oberst Patient Satisfaction Scale, have been translated into other languages (Findik et al., 2010; Ozturk et al., 2020; Peršolja, 2018; Ríos‐Risquez & García‐Izquierdo, 2016; Tang et al., 2013). Nursing‐specific measures exist and are accessible, but they were used much less frequently (26.6%) in this review.

### 5.2 Limitations

Most of the articles included in this review reported on cross‐sectional, qualitative or quasi‐experimental studies. These study designs limited the conclusions authors could draw. However, our rigourous application of the integrative review allowed us to identify elements of patient satisfaction as described in these studies. There is an underrepresentation in the literature of psychiatric care. In fact, many studies excluded psychiatric patients from their participant populations. This exclusion limits our ability to understand all elements of patient satisfaction. Another limitation is that many of the articles included were from a medical or administrative perspective, not a nursing perspective, strictly speaking. Consequently, a number of elements identified as part and parcel of the concept of patient satisfaction were not necessarily associated with the work of nursing. Although the concept of patient satisfaction is not a nursing concept per se, nursing has embraced it. Our ability to identify elements of patient satisfaction essential to the work of nursing is a strength.

## 6 CONCLUSIONS

With the identification of 68, often contradictory, elements of patient satisfaction and the presence of 22 discrete measures within the included studies, patient satisfaction is a difficult‐to‐pin‐down concept. This is concerning because of the increased weight patient satisfaction has on nursing evaluation and dispersal of funds for patient care, not just in the United States but around the world. Nursing researchers in the United States rely heavily on HCAHPS and Press Ganey. Nations outside the United States use a variety of different measures, only six of which heavily involve nursing elements.

Nurses also need to be aware that an emphasis on patient satisfaction should not take their focus away from high‐quality, individualized patient care. Because patient satisfaction will contribute to nursing performance measures and creation of nursing policy for the foreseeable future, a nursing‐specific definition is needed. Definitional elements of patient satisfaction specific to nursing care must be identified and matching instruments developed.

The nursing community must come together to create a nursing‐specific measure of patient satisfaction. This measure would emphasize aspects of patient satisfaction at the interpersonal exchange between nurse and patient where nursing communication, responsiveness and accountability can have an impact. Patient‐Reported Outcomes Measurement Information System **(**PROMIS©) is a programme developed to validate measures of patient‐reported outcomes. Currently, there is no measure listed in PROMIS© for patient satisfaction (Health measures: Northwestern University, 2021). If nurses are to be evaluated using patient satisfaction measures, the PROMIS© methodology can guide development of that measure and make it available for use throughout the world.

## AUTHOR CONTRIBUTIONS

GG, ML: Study design. GG: Data collection. GG, ML: Data analysis. ML: Study supervision. GG, ML: Manuscript writing. GG, ML: Critical revisions for important intellectual content.

## CONFLICT OF INTEREST

The authors declare no conflicts of interest.

## Supporting information


Table S1

Table S2

Table S3

## References

[nop21437-bib-0001] Abdellah, F. G. , & Levine, E. (1957). Developing a measure of patient and personnel satisfaction with nursing care. Nursing Research, 5(3), 100–108. 10.1097/00006199-195702000-00002 13400373

[nop21437-bib-0002] Aiken, L. H. , Sermeus, W. , van den Heede, K. , Sloane, D. M. , Busse, R. , McKee, M. , Bruyneel, L. , Rafferty, A. M. , Griffiths, P. , Moreno‐Casbas, M. T. , Tishelman, C. , Scott, A. , Brzostek, T. , Kinnunen, J. , Schwendimann, R. , Heinen, M. , Zikos, D. , Sjetne, I. S. , Smith, H. L. , & Kutney‐Lee, A. (2012). Patient safety, satisfaction, and quality of hospital care: Cross sectional surveys of nurses and patients in 12 countries in Europe and the United States. BMJ: British Medical Journal (Clinical Research Edition), 344, e1717. 10.1136/bmj.e1717 PMC3308724 22434089

[nop21437-bib-0003] Audi, R. (Ed.) (2015). Definition. In The Cambridge dictionary of philosophy (pp. 247–249). Cambridge University Press.

[nop21437-bib-0004] Bernays, E. L. (1947). What patients say about nurses. The American Journal of Nursing, 47, 93–96.

[nop21437-bib-0005] Boulding, W. , Glickman, S. W. , Manary, M. P. , Schulman, K. A. , & Staelin, R. (2011). Relationship between patient satisfaction with inpatient care and hospital readmission within 30 days. American Journal of Managed Care, 17(1), 41–48. 21348567

[nop21437-bib-0006] Burns‐Bolton, L. , Aydin, C. E. , Donaldson, N. , Brown, D. S. , Nelson, M. S. , & Harms, D. (2003). Nurse staffing and patient perceptions of nursing care. JONA: The Journal of Nursing Administration, 33(11), 607–614. 10.1097/00005110-200311000-00011 14608220

[nop21437-bib-0007] Butler, R. , Monsalve, M. , Thomas, G. W. , Herman, T. , Segre, A. M. , Polgreen, P. M. , & Suneja, M. (2018). Estimating time physicians and other health care workers spend with patients in an intensive care unit using a sensor network. The American Journal of Medicine, 131(8), 972.e9–972.e15. 10.1016/j.amjmed.2018.03.015 29649458

[nop21437-bib-0008] Centers for Medicare and Medicaid Services . (2020a). Hospital CAHPS (HCAHPS) . https://www.cms.gov/Research‐Statistics‐Data‐and‐Systems/Research/CAHPS/HCAHPS1

[nop21437-bib-0009] Centers for Medicare and Medicaid Services . (2020b). Quality Assurance Guidelines – HCAHPS . https://hcahps.org/globalassets/hcahps/quality‐assurance/2020_qag_v15.0.pdf

[nop21437-bib-0010] Chen, Q. , Beal, E. W. , Okunrintemi, V. , Cerier, E. , Paredes, A. , Sun, S. , Olsen, G. , & Pawlik, T. M. (2018). The association between patient satisfaction and patient‐reported health outcomes. Journal of Patient Experience, 6(3), 201–209. 10.1177/2374373518795414 31535008 PMC6739681

[nop21437-bib-0011] Copp, L. A. (1971). The psychology of patient satisfaction. Bedside Nurse, 4, 23–26. 5205221

[nop21437-bib-0012] Donabedian, A. (1992). The Lichfield lecture. Quality Assurance in health care: Consumer's role. Quality and Safety in Health Care, 1(4), 247–251. 10.1136/qshc.1.4.247 PMC1055035 10136873

[nop21437-bib-0013] Donabedian, A. (1966). Evaluating the quality of medical care. The Milbank Memorial Fund Quarterly, 44(3), 166–206. 10.1111/j.1468-0009.2005.00397.x 5338568

[nop21437-bib-0014] Donabedian, A. (1988). The quality of care: How can it be assessed? JAMA: The Journal of the American Medical Association, 260(12), 1743–1748. 10.1001/jama.1988.03410120089033 3045356

[nop21437-bib-0015] Dozier, A. M. , Kitzman, H. J. , Ingersoll, G. L. , Holmberg, S. , & Schultz, A. W. (2001). Development of an instrument to measure patient perception of the quality of nursing care. Research in Nursing & Health, 24(6), 506–517. 10.1002/nur.10007 11746079

[nop21437-bib-0016] Dragovich, A. , Beltran, T. , Baylor, G. M. , Swanson, M. , & Plunkett, A. (2017). Determinants of patient satisfaction in a private practice pain management clinic. Pain Practice, 17(8), 1015–1022. 10.1111/papr.12554 28083999

[nop21437-bib-0017] Fenton, J. J. , Jerant, A. F. , Bertakis, K. D. , & Franks, P. (2012). The cost of satisfaction: A national study of patient satisfaction, health care utilization, expenditures, and mortality. Archives of Internal Medicine, 172(5), 405–411. 10.1001/archinternmed.2011.1662 22331982

[nop21437-bib-0018] Findik, U. Y. , Unsar, S. , & Sut, N. (2010). Patient satisfaction with nursing care and its relationship with patient characteristics. Nursing & Health Sciences, 12(2), 162–169. 10.1111/j.1442-2018.2009.00511.x 20602687

[nop21437-bib-0019] Flood, R. , Szwargulski, P. , Qureshi, N. , Bixby, M. , Laffey, S. , Pratt, R. , & Gerard, J. (2016). Immediate bedding and patient satisfaction in a pediatric emergency department. Journal of Emergency Medicine, 50(5), 791–798. 10.1016/j.jemermed.2015.10.008 26577525

[nop21437-bib-0020] Foss, C. , & Hofoss, D. (2004). Patients' voices on satisfaction: Unheeded women and maltreated men? Scandinavian Journal of Caring Sciences, 18(3), 273–280. 10.1111/j.1471-6712.2004.00290.x 15355521

[nop21437-bib-0021] Godley, M. , & Jenkins, J. B. (2019). Decreasing wait times and increasing patient satisfaction: A lean six sigma approach. Journal of Nursing Care Quality, 34(1), 61–65. 10.1097/NCQ.0000000000000332 29889720

[nop21437-bib-0022] Gray, E. , Santiago, L. , Dimalanta, M. , Maxton, J. , & Aronow, H. (2016). Discharge by 11:00 a.m.: The significance of discharge planning. Medsurg Nursing, 25(6), 381–384. 30304602

[nop21437-bib-0023] Health Measures: Northwestern University . (2021). PROMIS. Health measures . https://www.healthmeasures.net/explore‐measurement‐systems/promis

[nop21437-bib-0024] Hekkert, K. D. , Cihangir, S. , Kleefstra, S. M. , van den Berg, B. , & Kool, R. B. (2009). Patient satisfaction revisited: A multilevel approach. Social Science & Medicine, 69(1), 68–75. 10.1016/j.socscimed.2009.04.016 19446942

[nop21437-bib-0025] Hendriks, A. A. J. , Vrielink, M. R. , Smets, E. M. A. , van Es, S. Q. , & De Haes, J. C. J. M. (2001). Improving the assessment of (in)patients' satisfaction with hospital care. Medical Care, 39(3), 270–283. 11242321 10.1097/00005650-200103000-00007

[nop21437-bib-0026] Kennedy, G. D. , Tevis, S. E. , & Kent, K. C. (2014). Is there a relationship between patient satisfaction and favorable outcomes? Annals of Surgery, 260(4), 592–600. 10.1097/SLA.0000000000000932 25203875 PMC4159721

[nop21437-bib-0027] Kessler, D. P. , & Mylod, D. (2011). Does patient satisfaction affect patient loyalty? International Journal of Health Care Quality Assurance, 24(4), 266–273. 10.1108/09526861111125570 21938972

[nop21437-bib-0028] Kurowski, B. , & Shaughnessy, P. (1982). The measurement and assurance of quality. In R. Vogel & H. Palmer (Eds.), Long‐term care: Perspectives from research and demonstrations (pp. 103–133). USDHHS Health Care Financing Administration.

[nop21437-bib-0029] Larrabee, J. , Ostrow, C. , Withrow, M. , Janney, M. , Hobbs, G. , & Burant, C. (2004). Predictors of patient satisfaction with inpatient hospital nursing care. Research in Nursing & Health, 27(4), 254–268. 10.1002/nur.20021 15264264

[nop21437-bib-0030] Larsson, G. , & Wilde‐Larsson, B. (2010). Quality of care and patient satisfaction: a new theoretical and methodological approach. International Journal of Health Care Quality Assurance, 23(2), 228–247. 10.1108/09526861011017120 21388102

[nop21437-bib-0031] McGhee, A. (1961). The patient's attitude to nursing care. Livingstone.

[nop21437-bib-0032] Mohammadipour, F. , Atashzadeh, S. F. , Parvizy, S. , & Hosseini, M. (2017). An explanatory study on the concept of nursing presence from the perspective of patients admitted to hospitals. Journal of Clinical Nursing, 26(23–24), 4313–4324. 10.1111/jocn.13758 28178371

[nop21437-bib-0033] Mrayyan, M. T. (2006). Jordanian nurses' job satisfaction, patients' satisfaction and quality of nursing care. International Nursing Review, 53(3), 224–230. 10.1111/j.1466-7657.2006.00439.x 16879186

[nop21437-bib-0034] Nguyen, T. V. F. , Bosset, J. F. , Monnier, A. , Fournier, J. , Perrin, V. , Baumann, C. , Brédart, A. , & Mercier, M. (2011). Determinants of patient satisfaction in ambulatory oncology: A cross sectional study based on the OUT‐PATSAT35 questionnaire. BMC Cancer, 11(1), 526. 10.1186/1471-2407-11-526 22204665 PMC3317877

[nop21437-bib-0035] Ozturk, H. , Demirsoy, N. , Sayligil, O. , & Florczak, K. L. (2020). Patients' perceptions of nursing care in a university hospital. Nursing Science Quarterly, 33(1), 12–18. 10.1177/0894318419881798 31795894

[nop21437-bib-0036] Peršolja, M. (2018). The effect of nurse staffing patterns on patient satisfaction and needs: A cross‐sectional study. Journal of Nursing Management, 26(7), 858–865. 10.1111/jonm.12616 30171648

[nop21437-bib-0037] Petrullo, K. , Lamar, S. , Nwankwo‐Otti, O. , Alexander‐Mills, K. , & Viola, D. (2012). The patient satisfaction survey: What does it mean to your bottom line? Journal of Hospital Administration, 2(2), 1. 10.5430/jha.v2n2p1

[nop21437-bib-0038] Polak, P. , Świątkiewicz‐Mośny, M. , & Wagner, A. (2019). Much Ado about nothing? The responsiveness of the healthcare system in Poland through patients' eyes. Health Policy, 123(12), 1259–1266. 10.1016/j.healthpol.2019.09.011 31635857

[nop21437-bib-0039] Press Ganey . (2021). Patient experience . https://www.pressganey.com/solutions/patient‐experience

[nop21437-bib-0040] Reichheld, F. F. (2003). The one number you need to grow (cover story). Harvard Business Review, 81(12), 46–54. 14712543

[nop21437-bib-0041] Ríos‐Risquez, M. I. , & García‐Izquierdo, M. (2016). Patient satisfaction, stress and burnout in nursing personnel in emergency departments: A cross‐sectional study. International Journal of Nursing Studies, 59, 60–67. 10.1016/j.ijnurstu.2016.02.008 27222451

[nop21437-bib-0043] Sjetne, I. S. , Veenstra, M. , & Stavem, K. (2007). The effect of hospital size and teaching status on patient experiences with hospital care: A multilevel analysis. Medical Care, 45(3), 252–258. 10.1097/01.mlr.0000252162.78915.62 17304083

[nop21437-bib-0044] Suhonen, R. , Papastavrou, E. , Efstathiou, G. , Tsangari, H. , Jarosova, D. , Leino‐Kilpi, H. , Patiraki, E. , Karlou, C. , Balogh, Z. , & Merkouris, A. (2012). Patient satisfaction as an outcome of individualised nursing care. Scandinavian Journal of Caring Sciences, 26(2), 372–380. 10.1111/j.1471-6712.2011.00943.x 22070423

[nop21437-bib-0045] Tang, W. , Soong, C. , & Lim, W. (2013). Patient satisfaction with nursing care: A descriptive study using interaction model of client health behavior. International Journal of Nursing Science, 3(2), 51–56. 10.5923/j.nursing.20130302.04

[nop21437-bib-0046] Turris, S. A. (2005). Unpacking the concept of patient satisfaction: A feminist analysis. Journal of Advanced Nursing, 50(3), 293–298. 10.1111/j.1365-2648.2005.03392.x 15811108

[nop21437-bib-0047] Westaway, M. S. , Rheeder, P. , Van Zyl, D. G. , & Seager, J. R. (2003). Interpersonal and organizational dimensions of patient satisfaction: The moderating effects of health status. International Journal for Quality in Health Care, 15(4), 337–344. 12930049 10.1093/intqhc/mzg042

[nop21437-bib-0048] Westbrook, K. W. , Babakus, E. , & Grant, C. C. (2014). Measuring patient‐perceived hospital service quality: Validity and managerial usefulness of HCAHPS scales. Health Marketing Quarterly, 31(2), 97–114. 10.1080/07359683.2014.907114 24878401

[nop21437-bib-0049] Whittemore, R. , & Knafl, K. (2005). The integrative review: Updated methodology. Journal of Advanced Nursing, 52(5), 546–553. 10.1111/j.1365-2648.2005.03621.x 16268861

[nop21437-bib-0050] Wolf, J. A. , Niederhauser, V. , Marshburn, D. , & LaVela, S. (2014). Defining patient experience. Patient Experience Journal, 1(1), 7–19.

[nop21437-bib-0051] Yen, M. , & Lo, L. (2004). A model for testing the relationship of nursing care and patient outcomes. Nursing Economic$, 22(2), 75–80. 15108476

