# Supplementary material for: Elements of patient satisfaction: An integrative review
Source: Nurs Open. 2022 Oct 28;10(3):1258–69. doi: 10.1002/nop2.1437 (PMC9912404; doi:10.1002/nop2.1437)
Supplement: Supplementary file 1 — Table S1 [file NOP2-10-1258-s002.docx]

**Supplemental Material**

**Supplemental Table 1**

*Definitions of Patient Satisfaction*

| Definition of Patient Satisfaction | Author and Year | Cited in Article By: |
| --- | --- | --- |
| The degree of convergence between the expectations that patients have of ideal care and their perceptions of the care that they actually receive. | Risser, 1975 | Mravyan, 2006  Findik et al., 2010 |
| The recipient’s reaction to salient aspects of the context, process and result of the service experience. | Pascoe, 1983 | Suhonen et al., 2012 |
| A personal evaluation of health care services and providers | Sitza & Wood, 1997 | Findik et al., 2010 |
| A response to nurse caring | Sherwood, 1997 | Larrabee, 2003 |
| A measure of the status of the interaction between healthcare professionals and service users | Hendriks et al., 2001 | Rìos-Risquez & Garcìa-Izquierdo, 2016 |
