# Supplementary material for: Elements of patient satisfaction: An integrative review
Source: Nurs Open. 2022 Oct 28;10(3):1258–69. doi: 10.1002/nop2.1437 (PMC9912404; doi:10.1002/nop2.1437)
Supplement: Supplementary file 2 — Table S2 [file NOP2-10-1258-s001.docx]

**Supplemental Table 2**

*Instruments Used to Measure Patient Satisfaction*

| **# of studies** | **Instrument** | **Authors** |
| --- | --- | --- |
| 6 | HCAHPS | Kennedy at al., 2013; Aiken et al., 2012; Westbrook et al., 2014; Boulding et al, 2011; Fenton et al, 2012; Elliott et al, 2008 |
| 3 | Press Ganey Patient Satisfaction scores | Flood et al, 2016; Godley & Jenkins, 2019; Kessler & Mylod, 2011 |
| 3 | Patient Perception of Hospital Experience in Nursing (PPHEN) † | Peršolja, 2018; Ozturk et al., 2019; Dozier et al., 2001 |
| 1 | Patient Satisfaction Scale (PSS) | Suhonen et al, 2012 |
| 1 | Patients’ Evaluation of Performance in California project (PEP-C) | Burns-Bolton et al, 2001 |
| 1 | Self-made scale | Westway et al., 2003 |
| 1 | Norwegian national patient satisfaction survey | Foss & Hofoss, 2004 |
| 1 | Patients’ Judgments of Nursing Care† | Larrabee et al., 2004 |
| 1 | The Patient Assessment of Hospital Care (PAHC) | Yen & Lo, 2004 |
| 1 | The Satisfaction with Nursing Care† | Mrayyan, 2006 |
| 1 | Patient Experience Questionnaire (PEQ) | Sjetne, Veenstra, & Satvem, 2007 |
| 1 | Core Questionnaire for Patient Satisfaction (COPS, In Dutch) | Hekkert et al, 2009 |
| 1 | Care-context adapted version of the (emotional stress reaction questionnaire) ESRQ | Larsson & Wild-Larsson, 2010 |
| 1 | Newcastle Satisfaction with Nursing Care Scales (NSNCS)† | Findik et al., 2010 |
| 1 | OUT-PATSAT35 | Nguyen et al, 2011 |
| 1 | Patient Satisfaction with Nursing Care Scale (PSNCS)† | Tang et al., 2013 |
| 1 | La Monica-Oberst Patient Satisfaction Scale (Spanish version) † | Rìos-Risquez & Garcìa-Izquierdo, 2016 |
| 1 | Army Provider Level Satisfaction Scale (APPLS) (adapted) | Dragovich et al., 2017 |
| 1 | MEPS-HC | Chen et al., 2019 |
| 1 | *Centrum Badania Opinii Społecznej* (CBOS) survey (Polish) | Polak et al., 2019 |
| 1 | Interview (qualitative study) | Mohammadipour et al., 2017 |

† Nursing-Specific Measures of Patient Satisfaction
