# Supplementary material for: Elements of patient satisfaction: An integrative review
Source: Nurs Open. 2022 Oct 28;10(3):1258–69. doi: 10.1002/nop2.1437 (PMC9912404; doi:10.1002/nop2.1437)
Supplement: Supplementary file 3 — Table S3 [file NOP2-10-1258-s003.docx]

**Supplemental Table 3**

*Patient Satisfaction Outcomes*

| # | **Relationship** | **Article** | **Country** |
| --- | --- | --- | --- |
| 1 | Patient Satisfaction effect on patient loyalty (small magnitude but statistically significant) | Kessler & Mylod, 2011 | USA |
| 2 | Patient satisfaction effect on 30-day hospital readmission (negative relationship) | Boulding et al, 2011 | USA |
| 3 | Patient Satisfaction related to lower odds of ED visits over 1 year | Fenton et al, 2012 | USA |
| 4 | Patient satisfaction related to higher odds of inpatient admission over 1 year. | Fenton et al, 2012 | USA |
| 5 | Patient Satisfaction related to greater healthcare expenditures over 1 year | Fenton et al, 2012 | USA |
| 6 | Patient Satisfaction related to greater prescription medication expenditures over 1 year | Fenton et al, 2012 | USA |
